# Exosome-Mediated Response to Cancer Therapy: Modulation of Epigenetic Machinery

**DOI:** 10.3390/ijms23116222

**Published:** 2022-06-02

**Authors:** Mohammad Imran Khan, Reem K. M. E. Alsayed, Hani Choudhry, Aamir Ahmad

**Affiliations:** 1 Biochemistry Department, Faculty of Science, King Abdulaziz University, Jeddah 21589, Saudi Arabia; mikhan@kau.edu.sa (M.I.K.); hchoudhry@kau.edu.sa (H.C.); 2 Centre of Artificial Intelligence for Precision Medicines, King Abdulaziz University, Jeddah 21589, Saudi Arabia; 3 Translational Research Institute, Academic Health System, Hamad Medical Corporation, Doha 3050, Qatar; ralsayed2@hamad.qa

**Keywords:** cancer drug resistance, exosomes, miRNAs, lncRNAs, methyltransferases

## Abstract

Exosomes, the extracellular vesicles produced in the endosomal compartments, facilitate the transportation of proteins as well as nucleic acids. Epigenetic modifications are now considered important for fine-tuning the response of cancer cells to various therapies, and the acquired resistance against targeted therapies often involves dysregulated epigenetic modifications. Depending on the constitution of their cargo, exosomes can affect several epigenetic events, thus impacting post-transcriptional regulations. Thus, a role of exosomes as facilitators of epigenetic modifications has come under increased scrutiny in recent years. Exosomes can deliver methyltransferases to recipient cells and, more importantly, non-coding RNAs, particularly microRNAs (miRNAs), represent an important exosome cargo that can affect the expression of several oncogenes and tumor suppressors, with a resulting impact on cancer therapy resistance. Exosomes often harbor other non-coding RNAs, such as long non-coding RNAs and circular RNAs that support resistance. The exosome-mediated transfer of all this cargo between cancer cells and their surrounding cells, especially tumor-associated macrophages and cancer-associated fibroblasts, has a profound effect on the sensitivity of cancer cells to several chemotherapeutics. This review focuses on the exosome-induced modulation of epigenetic events with resulting impact on sensitivity of cancer cells to various therapies, such as, tamoxifen, cisplatin, gemcitabine and tyrosine kinase inhibitors. A better understanding of the mechanisms by which exosomes can modulate response to therapy in cancer cells is critical for the development of novel therapeutic strategies to target cancer drug resistance.

## 1. Cancer Therapy and Drug Resistance

Chemotherapeutic drugs aim to kill cancer cells through the disruption of mechanisms that tumor cells rely on for survival, proliferation or migration [1]. They target the synthesis and/or function of proteins or nucleic acids that are involved in mechanisms contributing to cancer progression. By debilitating the cancer cell’s ability to function optimally for survival and tumorigenesis, chemotherapeutic agents eventually result in the death of cancer cells. Often, chemotherapy is employed in adjuvant settings along with surgery, radiation therapy and even with other chemotherapeutic drugs [2]. Despite the many benefits of chemotherapy for cancer cure, the associated toxicity is also well known [3]. This is because chemotherapeutic drugs target rapidly dividing cells and can thus impact several other cells that constantly divide, such as, hair follicles, bone marrow, cells of the gastrointestinal tract, etc. [1]. Another major challenge with the use of chemotherapy is the phenomenon of acquired resistance [4,5]. The drug resistance that develops as a result of continuous administration of chemotherapy has a significant impact on the clinical outcome of cancer patients.

Drug resistance, or tachyphylaxis, in cancer has long been a subject of investigation and a number of underlying mechanisms have been proposed [6,7,8]. Primarily, there are two types of resistance, primary resistance wherein the patient is inherently resistant to the drug even before the drug administration, and secondary (acquired) resistance wherein, the resistance is ‘acquired’ post-drug administration [9]. Efflux pumps, the active transporters on cell surface, pump out the drugs from the cells through the production of p-glycoprotein [1] while multidrug resistance-associated protein 1, multidrug resistance protein 1 and breast cancer resistance protein are examples of transporters that also mediate drug resistance in many different cancers, with tyrosine kinase inhibitors (TKIs) being their well-known targets [10]. Often the chemotherapeutic drugs require activation to be functional and effective, and the drug resistance could be induced through the inhibition of drug activation [10]. Additionally, for an effective targeting of cancer cells, molecular targets must be available within the cancer cell and downregulation or mutations in these molecular targets could lead to drug resistance. Since some chemotherapies induce DNA damage leading to apoptosis, DNA damage repair mechanisms can also induce resistance [10]. For example, cisplatin, a platinum-based drug, creates DNA crosslinks thus impacting DNA replication and cell division but cancer cells circumvent these effects through increased DNA repair. Furthermore, cancer cells manipulate autophagy and the apoptosis pathways to inhibit cell death, again resulting in resistance to drugs [8,11]. The genetic makeup of the patient [8] as well as the process of epithelial to mesenchymal transition (EMT) [12] also play a role in the acquisition of resistance. It has been observed that the drug-resistant cancer cells in a tumor outlive the drug-sensitive cells that are being targeted by the drug. Therefore, drug resistance increases and dominates over time with the increase in proportion of resistant cells within a progressing tumor.

A number of chemotherapeutic agents are FDA approved for cancer treatment but some of the drugs that we discuss later in this article include tamoxifen, cisplatin, gemcitabine and TKIs. Tamoxifen is a competitive inhibitor of estrogen, and it binds the estrogen receptor alpha on ER+ breast cancer cells [13]. However, resistance to tamoxifen is observed due to the upregulation of growth factor signaling, as growth factors activate kinases that phosphorylate estrogen receptors to be constitutively active. Hence, there is an alteration in the drug target that debilitates the drug. As mentioned earlier, cisplatin is a platinum-based drug that induces DNA damage in many different cancers [14,15,16]. The resistance against cisplatin is acquired through multiple pathways and these include DNA damage repair, dysregulation of apoptosis, inactivation of cisplatin and reduced cellular accumulation [14]. Gemcitabine is a chemotherapeutic drug that is used against a variety of solid tumors to induce apoptosis [17]. It requires phosphorylation to be active and incorporation into the newly synthesized DNA strands in dividing cells, thus interfering with DNA synthesis. An alteration in the transporters that uptake gemcitabine or an inhibition of factors responsible for phosphorylation could lead to gemcitabine resistance. Finally, TKIs have been used to treat various cancers through their targeting of signal transduction pathways that require receptor tyrosine kinases [18]. Multiple generations of TKIs are now being used and the very need to come up with these successive TKI generations was due to the development of resistance against the preceding generation(s). Mutations in the drug targets (receptor tyrosine kinases) that alter the kinase binding domain are the main route of resistance against TKIs [19]. It cannot be emphasized enough that resistance against therapies, particularly the chemotherapeutic drugs, is a major clinical challenge necessitating the validation of novel diagnostic, prognostic as well as therapeutic targets.

## 2. Epigenetic Cargo of Exosomes

For past several years, the role of exosomes is being investigated in relation to the acquired resistance against chemotherapeutic drugs [20]. Exosomes are extracellular vesicles that package and transport proteins and nucleic acids from cells to their nearby cells [21]. They have an average size of approximately 100 nm and their functions include cellular homeostasis [22,23] and intercellular communication [24]. Exosomes can exert a multitude of biological functions, often conflicting, based on the composition of their cargo. Some exosomes can induce apoptosis, others can promote survival while others can still modulate the immune response [21]. On the virtue of their cargo, exosomes have also been proposed as potential cancer diagnostic and/or prognostic biomarkers [25]. Additionally, efforts are underway to possibly deploy exosomes as drug delivery systems as part of the anti-cancer therapy [20]. Exosomes modulate drug resistance through several mechanisms. They can trick the drugs by carrying and presenting receptors that are drug targets, so as to attract drugs off the cancer cells. Exosomes can also transfer the ‘characteristics’, i.e., the factors (signaling proteins, coding and non-coding RNAs, etc.) that mediate resistance. In particular, studies over the past several years have detailed the role of exosomes in the transfer of epigenetic machinery that includes methyltransferases as well as non-coding RNAs, resulting in epigenetic regulation of cancer drug resistance. This section details the current knowledge on the epigenetic cargo of exosomes that determines the sensitivity/resistance of tumor cells to different therapies.

### 2.1. Exosome-Mediated Transfer of DNA Methyltransferases

An important mechanism by which exosomes can epigenetically regulate drug resistance is through the transfer of methyltransferases. In a study [26] that focused on developing a new technology—a sensitive microfluidic platform for on-chip analysis of mRNA contents of exosomes—it was found that DNA methyltransferase MGMT (O-6-methylguanine DNA methyltransferase) mRNA is not only detectable in exosomes, but the levels are reflective of changes in the parental tumor cells. The proof-of-principle study was conducted in a glioma model with the background that since successive biopsies of glioma patients are challenging, there is need to develop alternative methods for evaluating disease progression, particularly drug resistance. In this context, it was suggested that exosomes from the patients’ blood might be exploited for the evaluation of their cargo content. MGMT is a DNA repair enzyme [27] and therefore involved in acquired cancer drug resistance. MGMT’s promoter is methylated in cells that are sensitive to drug treatment. Accordingly, it was found that the parental glioma cells that were sensitive to temozolomide (TMZ) had lower MGMT mRNA levels which corresponded with higher sensitivity to TMZ [26]. The exosomes derived from such cells also had relatively lower levels of MGMT mRNA. On the other hand, TMZ-resistant glioma cancer cells had elevated levels of MGMT that probably played a role in the TMZ resistance but more importantly, the higher levels of MGMT were also found in the exosomes derived from such resistant cells. In addition to the initial in vitro studies in the glioma cancer cells, the study further tested blood samples from glioma patients. It was reported that exosomal MGMT levels in samples derived from methylation-positive patients were comparable to those from the healthy controls [26]. Such patients would be expected to be sensitive to chemotherapy. In contrast, glioma patients with lower MGMT promoter DNA methylation had higher exosomal MGMT [26]. These are the patients at risk of demonstrating acquired resistance against chemotherapy.

While the above study [26] demonstrated exosomal presence of DNA methyltransferase, the manifestations of such phenomenon in recipient cells were not evaluated. It would not be wrong to expect that exosomal transfer of factors responsible for acquired drug resistance should result in enhanced resistance of exosome-receiving cells against chemotherapy. This was demonstrated in a later study by another group that studied cisplatin resistance in ovarian cancer [28]. First, in agreement with the earlier study on TMZ resistance in glioma [26], this study [28] also found a positive correlation between methyltransferase DNA methyltransferase 1 transcripts in cancer cells and the exosomes that originated from those cells. Furthermore, this study also showed that the cells receiving exosomes, through co-culture, acquire resistance against cisplatin, thus clearly establishing a role of exosomal transfer of methyltransferases in acquired drug resistance. In vivo testing was also carried out and it was reported that the administration of DNA methyltransferase 1-harboring exosomes significantly increased tumor growth in vivo [28]. To rule out factors other than exosomes playing a role in the acquired resistance, exosome inhibitor GW4869 was used in this study to demonstrate that exosome inhibition was enough to reverse the phenomenon of acquired drug resistance.

Subsequent to this study demonstrating an effect of exosomal methyltransferases on acquired cancer drug resistance [28], another independent research team evaluated such effect of exosomal MGMT in glioma TMZ resistance. The focus of this study was on cellular interactions within the tumor microenvironment, particularly cancer cells’ interactions with astrocytes [29]. Firstly, glioma cancer cells could stimulate astrocytes into ‘reactive’ astrocytes and the exosomal cargo from these reactive astrocytes had particularly high levels of MGMT [29] compared to exosomes from normal astrocytes. Furthermore, as evidence to support the ability of exosome-derived methyltransferases to induce resistance against chemotherapy, it was found that MGMT-negative glioma cells (TMZ-sensitive glioma cells with promoter methylated MGMT) could take up exosomes from reactive astrocytes (with high MGMT) and demonstrate resistance against TMZ [29]. Thus, combined with the knowledge gained from an earlier study [26], it is evident that MGMT transportation in exosomes plays an important role in glioma TMZ resistance. It is now also being realized that promoter methylation is not the only factor driving MGMT expression, but MGMT genomic rearrangements also have a fair share in determining MGMT expression with the evidence of such MGMT genomic rearrangements in recurrent gliomas leading to MGMT overexpression [30]. This information is relevant to MGMT’s role in glioma cell TMZ resistance. Additionally, such fusions were also detected in glioma-derived exosomes [30] which further indicates their exosomal transport as a possible mechanism of spreading drug resistance within the microenvironment. This also opens up a possible exploitation of exosome cargo for diagnosis of relapsed/recurrent disease.

Clearly, the studies discussed so far provide evidence for a role of methyltransferases in acquired cancer drug resistance of cancer cells and the transport of methyltransferases in the exosomal cargo with a resulting influence on the drug sensitivity/resistance of cells that receive the exosomes. On a different note, it has also been shown that exosomal cargo can modulate methyltransferases in the recipient cells with effects on drug resistance. This particular study focused not on the astrocytes but on another very important cell type within the tumor microenvironment—the fibroblasts—and it was shown that exosome-mediated transfer of miR-29b from cancer-associated fibroblasts (CAFs) to hepatocellular carcinoma (HCC) cells resulted in miR-29b-mediated suppression of DNA methyltransferase 3b in the HCC cells [31]. Thus, exosomes can carry cargo to effect methyltransferases in the recipient cells with resulting effects on their cellular phenotype.

As an indirect association of DNA methyltransferases and exosome cargo, the effects of an inhibitor decitabine were shown in colorectal cancer cells [32]. This study reported that the invasion potential of colorectal cancer cells with acquired resistance against oxaliplatin was significantly diminished when these cells were treated with decitabine. Although the transportation of any methyltransferase in exosomes was not evaluated in this study, the exosome cargo was checked for other epigenetic markers, the miRNAs, and it was found that miRNAs such as miR-200c and miR-141 were particularly elevated in the exosomes [32]. Both of these miRNAs belong to the miR-200 family of miRNAs and this miRNA family is involved in negative regulation of EMT [33]. Since the exosome cargo is reflective of levels in the cells that the exosomes are derived from, there was clear elevation of the epithelial marker—e-cadherin—in the cells treated with decitabine along with the elevated EMT-inhibiting miRNAs [32]. On a similar note, another class of non-coding RNAs, the circRNA (circular RNA), has also been shown to be affected by methylation in tumor cells. In this case, circCUX1 was methylated by methyltransferase-like 3 resulting in circCUX1 stabilization with resulting resistance against radiotherapy [34]. Yet, another type of non-coding RNAs, the long non-coding RNAs (lncRNAs), were also connected to methylation and exosomes in a study on multiple myeloma wherein two lncRNAs, LOC606724 and SNHG1, were reported to be elevated in multiple myeloma cells that were exposed to exosomes from adipocytes [35]. Methyltransferase-like 7A was found to mediate m^6^A methylation of lncRNAs and these lncRNAs were reported to correlate with poor prognosis of MM patients. 

### 2.2. Non-Coding RNAs as Exosomal Cargo

As briefly discussed in the preceding section, a number of non-coding RNAs are packaged inside the exosomes, and these include miRNAs, lncRNA and circRNAs. By far, miRNAs seem to be the most common non-coding RNAs that are packaged in exosomes [36] or at least these are the most studied non-coding RNA subtype in exosomes. miRNA loading into the exosomes depends on machinery that is specific and selective which, however, in cancer, is dysregulated resulting in omission or inclusion of specific miRNA(s) into the vesicles that promote tumor progression [36]. For example, in metastatic melanoma it was observed that the exosomes contained abnormally high pro-metastatic prominin-1 [36]. miRNA profiling revealed that among 49 over-expressed miRNAs in exosomes from metastatic melanoma, compared to those from normal cells, a total of 20 miRNAs associated with tumor progression. Exosomes package miRNAs that promote resistance to chemotherapy [37]. One pathway by which exosomes could induce resistance to drugs would be by packaging miRNAs that are responsible for initiating the EMT. This is something that was also indicated in the study involving decitabine, the methyltransferase inhibitor, discussed above [32]. Thus, exosomes not only carry miRNAs, but they might also even favor tumor- and resistance-promoting miRNAs. This is still debatable with no clear verdict and the exosomal cargo might just be reflective of miRNA content of the cells the exosomes were derived from. LncRNAs are another type of non-coding RNAs that regulate gene expression. They constitute the second most studied non-coding RNAs in the exosome, and the change in abundance of lncRNAs has been noted across tumor versus ‘normal’ exosomes [38]. The altered lncRNA cargo can have a possible effect on cancer hallmarks such as metastasis, angiogenesis and sensitivity to therapies. For example, in hepatocellular carcinoma cells, Linc-ROR inhibits chemotherapy-induces apoptosis and reduces drug cytotoxicity, and this lncRNA is found to be in high abundance in cancer cell exosomes versus normal cell exosomes [39]. In HER2+ breast cancer cells, exosomal LncRNA-SNHG14 has been shown to promote resistance [40]. In the next section, we provide more detailed account of exosomal miRNAs/lncRNA/circRNAs that have implicated in acquired resistance against specific chemotherapeutic agents in different human cancers.

## 3. Mechanisms of Exosome-Mediated Cancer Drug Resistance

### 3.1. Tamoxifen Resistance

Tamoxifen is used to target ER+ breast cancers, and a number of reports suggest that exosomes might play a role in the resistance against this drug. For example, exosomes that are rich in miR-22 are released from certain CAFs to induce tamoxifen resistance [41]. It is now known that tamoxifen resistance in ER+ breast cancer cells, such as MCF-7, correlates with the methylation status of oncogenes and the exosome cargo is responsible for the resistance against tamoxifen [42]. Tamoxifen resistance has also been observed to be induced by miR-221/222 in MCF-7 cells [43]. A significant distinction was found in the exosomal composition from wild type MCF-7 vs. tamoxifen-resistant MCF-7 cells. Exosomes from resistant cells induced resistance in parental MCF-7 cells by reducing the expression of p27 and Er-α genes. Tamoxifen-resistant MCF-7 cells can also transfer miR-9-5p, which reduces the expression of *ADIPOQ* *adiponectin, C1Q and collagen domain containing* gene hence inducing resistance to tamoxifen in neighboring cells [44]. *Adiponectin, C1Q and collagen domain containing* is a gene that codes adiponectin, which is a protein that, upon upregulation, contributes to increased survival and resistance against therapy. miR-181a-2 induces tamoxifen resistance in MCF-7 cells through exosome delivery, which is mechanistically mediated by the activation of the PI3K/Akt pathway and the inhibition of estrogen receptor signaling [45]. Another miRNA, miR-205, is released in exosomes by tamoxifen-resistant cells, leading to tamoxifen resistance in nearby cells through the downregulation of E2F Transcription Factor 1 in MCF-7 cells [20]. 

As mentioned above, miRNAs are not the only non-coding RNAs that mediate exosome-induced resistance against therapy in cancer cells. It was reported that in tamoxifen-resistant cells, the circular RNA circ_UBE2D2 is upregulated [46]. Upon packaging and transferring of circ_UBE2D2 through exosomes to other cells, resistance to tamoxifen was acquired through the interaction of circ_UBE2D2 with miR-200a-3p. This interaction between circular RNA and miRNA not only regulates resistance, but also cell viability and migration, which are the hallmarks of drug resistance. The lncRNA UCA1 was significantly more abundant in the exosomes of tamoxifen-resistant cells, compared to sensitive MCF-7 cells, and could induce resistance in the MCF-7 cells [47]. Another exosomal lncRNA, HOTAIR, was also found to highly correlate with tamoxifen resistance in breast cancer cells [48]. High HOTAIR levels led to poor disease-free as well as overall survival in breast cancer patients. Combined, a number of studies support a role of exosomes in facilitating tamoxifen resistance through the transport of various non-coding RNAs (Table 1).

### 3.2. Cisplatin Resistance

In several reports, exosomes have been observed to transfer cisplatin resistance through packaging epigenetic modifiers. In breast cancer cells MDA-MB-231, cisplatin-resistant cells transferred resistance through the transfer of miR-423-5p in the exosomes [50]. The expression of miR-423-5p highly impacted cisplatin resistance, as when the expression increased so did the resistance [22]. In lung cancer cells A549, cisplatin resistance is passed from one cell to the other, and this process is dependent on exosomal miR-100-5p [23]. Additionally, tumor-associated macrophages (TAMs) have been observed to deliver miR-21 to non-resistant/sensitive cancer cells, and this miRNA transforms gastric cancer cells to be cisplatin resistant [51]. miR-21 acts as an inducer of resistance through the activation of the PI3K/AKT signaling pathway and the suppression of PTEN expression, thereby inhibiting apoptosis [51]. In oral squamous cell carcinoma, exosomes have also been seen to package miR-21 and to confer resistance in drug-sensitive cancer cells through reducing DNA damage response to cisplatin and downregulating the expression of PTEN and PDCD4 [52]. PTEN and PDCD4 are known to have tumor-suppressing functions. While PTEN reduces tumor growth and affects PI3K/Akt/mTOR signaling pathway, PDCD4 is involved in many cellular functions and behaviors such as tumor growth, apoptosis, invasion and cell transformation. In osteosarcoma, the circular non-coding RNA hsa_circ_103801 was found to be overexpressed in cisplatin-resistant cells compared to the wide-type osteosarcoma. Exosomes containing hsa_circ_103801 not only suppressed apoptosis, but also promoted cisplatin resistance and increased the expression of resistance-promoting proteins [53]. 

After cisplatin treatment, CAFs increase the production and secretion of miR-522 into the exosomes, increasing cisplatin resistance in gastric cancer cells. Heterogeneous nuclear ribonucleoprotein A1 was the molecule chiefly responsible for the increase in miR-522 packaging in exosomes, and miR-522 modulated cisplatin resistance through the downregulation of the *arachidonate 15-lipoxygenase* gene and the reduction in lipid reactive oxygen species accumulation [26]. CAFs are also responsible for heterogeneous nuclear ribonucleoprotein A1-mediated aberrant packaging of miR-196a into exosomes to be delivered to head and neck cancer cells to modulate cisplatin resistance [54]. The way miR-196a functions to modulate resistance is through the inhibition of *cyclin-dependent kinase inhibitor 1B* and *inhibitor of growth family member 5* genes. The overexpression of *cyclin-dependent kinase inhibitor 1B* and *inhibitor of growth family member 5* would lead to the reduction in the growth of the cells and an increased sensitivity to chemotherapy, hence the inhibition of their expression through miR-196a leads to opposite effects, i.e., cisplatin resistance. In esophageal cancer, miRNA193 was seen to induce resistance through exosomal secretion, by its ability to inhibit the gene expression of the tumor suppressor gene *transcription factor AP-2 gamma* [55]. In yet another study, although the specific element in exosome was not identified, generally, exosomes from cisplatin-resistant HCC cancer cells conferred resistance to neighboring cells through the upregulation of P-glycoprotein [56]. Hence, through multiple pathways, exosomes deliver molecules that manipulate the epigenetics of the cells in a way that favors chemotherapeutic resistance of cisplatin (Table 2).

### 3.3. Gemcitabine Resistance

Gemcitabine resistance has also been demonstrated to be manipulated and transferred through exosome communication between cancer cells (Table 3). As discussed above, TAMs are known to release exosomes that aid in acquiring drug resistance. In pancreatic adenocarcinoma specifically, exosomes from TAMs deliver miR-365 to transform cancer cells to become gemcitabine resistant [59]. In native cells, miR-365 facilitates apoptosis, but in cancer cells, it contributes to cancer hallmarks. The gene target of miR-365 is nuclear factor I/B, and other functions of this miRNA include cell differentiation, metabolic regulation, increasing levels of triphospho nucleotides, and the upregulation in the metabolism of pyrimidine. Increase in triphospho nucleotide availability leads to the aberrant regulation of the cytidine deaminase, which is the principal enzyme contributing to gemcitabine inactivation and resistance. In non-small cell lung cancer (NSCLC), gemcitabine resistance can be transferred from cell to cell through exosomal miR-222-3p, which targets the promotor region of the SOCS3 gene [60]. Additionally, this miRNA also contributes to other hallmarks of cancer that increase aggressiveness, such as increased proliferation, migration and invasion. 

CAFs are inherently chemo resistant, and, upon exposing CAFs to gemcitabine, the levels of secreted transcription factor Snail, and its downstream effector miR-146a increase in the exosomes [63]. Additionally, the take up of these exosomes by pancreatic cancer cells also induces an increase in Snail and miR-146a expression, which leads to upregulation of CXCR4 gene expression, stimulation of NF-κB and Akt and upregulation of cancer stem cell markers, ultimately leading to enhanced chemoresistance, metastasis and cancer cell growth. Pancreatic cancer stem cells display a horizontal transfer of gemcitabine resistance through the delivery of overexpressed miR-210 via exosomes. The functions of miR-210 that render chemoresistance possible include the activation of mTOR signaling pathway, inhibition of cell cycle arrest and reduced apoptosis [64].

Another study on pancreatic cancer revealed a role of miR-106b in exosome-mediated gemcitabine resistance [62]. CAF-derived exosomes deliver miR-106b to pancreatic cancer cells where it carries out its function in targeting the gene *tumor protein P53 inducible nuclear protein 1* directly. Although the mechanisms of how it functions have not yet been uncovered, the *protein P53 inducible nuclear protein 1* gene is a known tumor suppressor in many different cancers, and its suppression through miR-106b leads to reduced sensitivity to gemcitabine. For pancreatic and ductal adenocarcinoma, gemcitabine resistance is also transferred between the cancer cells via exosomal miR-155 and miR-21 [61,65]. The way miR-155 functions in gemcitabine resistance is by preventing apoptosis through the stimulation of the anti-apoptotic pathway, downregulation of certain tumor suppressor genes such as *protein P53 inducible nuclear protein 1*/*Sel*-*1*-*like,* and upregulation of genes responsible for exosome synthesis. miR-21 is a well-characterized miRNA in terms of its role in determining resistance against multiple cancer therapies [66,67].

### 3.4. Resistance against TKIs

TKIs are inhibitors that bind to receptor tyrosine kinases and inhibit their function, and examples of these receptors include EGFR, AKT, c-MET, VEGFR and MEK1/2 [68]. It is only in recent few years that the exosome-mediated acquired resistance against several TKIs has been characterized in sufficient detail [69,70]. The acquired resistance against TKIs can also be affected by non-coding RNA cargo of exosomes. According to a study in renal cancer, the lncRNA lncARSR was found responsible for chemoresistance against the TKI sunitinib [71]. lncARSR performs its function of inducing resistance through miRNA sponging. This leads to the upregulation or higher activity of the miRNA targets, such as other receptor tyrosine kinases, namely, AXL and c-MET. In sunitinib-resistant cells, aberrant lncARSR expression leads to the aberrant expression of AXL and c-MET, which were found to be essential factors in modulating sunitinib resistance.

A number of TKIs, namely, imatinib, nilotinib and dasatinib, are widely used for the treatment of acute myeloid leukemia. Exosomes derived from human bone marrow microenvironment-derived mesenchymal stem cells upregulate the expression of Bcl-2 (an anti-apoptotic protein) and limit the activation of caspase-9, caspase-3 and Poly (ADP-ribose) polymerase cleavage. This epigenetic manipulation contributes to chemoresistance [72]. Plasminogen activator urokinase receptor (PLAUR) is a receptor that has a known function in cellular functions such as proliferation, cell motility and apoptosis. Exosomal PLAUR in NSCLC was observed to increase at mRNA as well as protein levels in gefitinib-resistant NSCLC cells [73]. Additionally, there was a positive high correlation between PLAUR and EGFR expression suggesting a role of exosomal PLAUR in gefitinib resistance, through the EGFR/p-AKT/survivin axis. Certain oncogenes that are responsible for lipogenesis are upregulated in human cancers, and cells utilize them to inhibit apoptosis and increase proliferation. Glycerol kinase 5 seems to play a role in gefitinib resistance of NSCLC through the inhibition of factors controlling lipogenesis [74]. The reduction in the glycerol kinase 5 protein levels in the exosomes leads to reduced levels of sterol regulatory element-binding transcription factor 1 and stearoyl-CoA desaturase-1, resulting in the attenuation of apoptosis inhibition in gefitinib-resistant cells.

The ability of exosomes, derived from TKI-resistant cancer cells, to induce TKI resistance in sensitive cells is now evident. As an example, exosomes from imatinib-resistant chronic myeloid leukemia K562 cells were shown to induce resistance in otherwise imatinib-sensitive K562 cells [75]. As evidence for the role of epigenetic cargo of exosomes in TKI resistance, exosomal miR-210 was shown to play a role in the resistance against third-generation TKI, osimertinib, in NSCLC cells [76]. miR-210 was found elevated in osimertinib-resistant HCC827 as well as PC-9 cells, relative to their native osimertinib-sensitive counterparts. Induction of EMT was found to be the underlying cause. In an independent study that also studied osimertinib resistance in NSCLC but used a different model system, osimertinib-sensitive H1975 vs. osimertinib-resistant H1975 cells, two exosome-derived miRNAs, miR-184 and miR-3913, stood out as the miRNAs that were elevated in osimertinib-resistant cells [77]. Interestingly, a proof-of-principle validation was carried out in three NSCLC patients that were administered osimertinib and from whom plasma was drawn, for exosome isolation, before and after the onset of osimertinib resistance. In renal cancer, miR-549a was found to be reduced and such lower levels were reflected in the derived exosomes that were taken up by endothelial cells and the reduced miR-549a levels resulted in de-repression of HIF-1α and VEGF ultimately leading to increased angiogenesis [78]. In support of an interplay between lncRNAs and the miRNAs, lncRNA LNC000093 was found to be downregulated while the miRNA it sponged, miR-675, is elevated in imatinib-resistant leukemia cells [79]. In summary, it is increasingly being realized that exosomes, through their epigenetic cargo, induce resistance against multiple TKIs in different human cancers (Table 4). 

## 4. Conclusions and Future Perspectives

**Figure 1:**
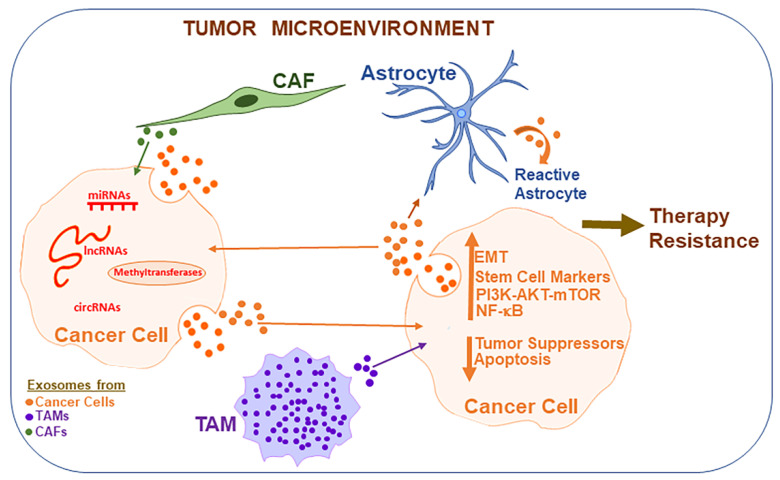
Exosome-mediated epigenetic regulation of cancer therapy resistance. Within the tumor microenvironment, various cell types communicate with each other leading to conditions favoring aggressive tumors and resistance against therapies, with exosomes playing an important role in cellular communications. Exosomes are released by various cell types and they transport epigenetic factors such as miRNAs, lncRNAs and circRNAs, in addition to carrying methyltransferases, all of which bring about epigenetic modifications in the recipient cells, resulting in the induction of EMT, cancer stem cell phenotype and cellular signaling pathways that favor cancer cell migration, invasion, angiogenesis, metastasis and therapy resistance. CAFs: cancer-associated fibroblasts, EMT: epithelial–mesenchymal transition, lncRNA: long non-coding RNA, miRNA: microRNA, mTOR: mammalian target of rapamycin, TAMs: tumor-associated macrophages.

Chemotherapeutics represent a major treatment strategy for clinical management of cancer patients. One of the most challenging aspects of cancer treatment is the acquired resistance against therapy i.e., acquisition of resistance against the very drug that is being administered. A better understanding of the underlying phenomena responsible for acquired cancer drug resistance is thus urgently needed. The existence of exosomes has been acknowledged for many years and they are well-known mediators of cell-to-cell communications, often transporting the proteins as well as nucleic acids between cancer cells and their surrounding cells, which could be the basis of acquired drug resistance. In this article, we presented evidence for the exosome-mediated transfer of epigenetic machinery-regulating factors (Figure 1). The methyltransferases, when transported by exosomes, can have a profound effect on gene expression in the recipient cells. Exosomes transport methyltransferases and may also impact the expression of methyltransferases in the recipient cells independent of a direct transport. Exosomes also transfer several non-coding RNAs, including miRNAs, lncRNA and circRNAs, within the tumor microenvironment, resulting in acquired therapy resistance. Clearly, information is still emerging and is far from complete. What is often lacking in the published reports is a complete mechanism by which an observed upregulated or downregulated factor/non-coding RNA can directly interfere with the anti-cancer drug action. Despite the lack of very conclusive knowledge, there seems to be enough evidence to support exosome-mediated regulation of epigenetic machinery in cancer and surrounding cells, resulting in conditions that favor therapy resistance. Design and conduct of such mechanism-focused studies will be key to the clinical future of exosomes in cancer research and treatment. Identification or synthesis of novel chemical compounds that can modulate exosome synthesis or function, along with novel non-toxic methods to deliver exosomes as therapeutics, can potentially rapidly advance the field. To this end, a close collaboration between cancer researchers, chemists and drug delivery experts will be highly desirable. Further investigations on the subject will be important to fully understand the role of exosomes in epigenetic drug resistance. This knowledge will invariably result in the identification and validation of invaluable cancer diagnostic as well as therapeutic targets.

## Figures and Tables

**Table 1:** Exosome-mediated tamoxifen resistance.

Exosomal Cargo	Non-Coding RNA Type	Exosomes Released From	Effect	Reference
circ_UBE2D2	Circular RNA	Cancer Cells	Interaction with miR-200a-3p	[46]
HOTAIR	lncRNA	Cancer Cells	Poor survival of patients	[48]
miR-9-5p	miRNA	Cancer Cells	Tamoxifen resistance	[44]
miR-22	miRNA	CAF	Tamoxifen resistance	[41]
miR-181a-2	miRNA	Cancer Cells	Activation of PI3K/Akt	[45]
miR-205	miRNA	Cancer Cells	E2F1 suppression	[49]
miR-221/miR-222	miRNA	Cancer Cells	p27 suppression	[43]
UCA1	lncRNA	Cancer Cells	Apoptosis inhibition	[47]

CAFs: cancer-associated fibroblasts, circRNA: circular RNA, E2F1: E2F Transcription Factor 1, lncRNA: long non-coding RNA, miRNA: microRNA.

**Table 2:** Exosome-mediated cisplatin resistance.

Exosomal Cargo	Non-Coding RNA Type	Cancer	Exosomes Released From	Effect	Reference
circ_103801	CircRNA	Osteosarcoma	Cancer Cells	Apoptosis suppression	[53]
miR-21	miRNA	Gastric	TAMs	Activation of PI3K/Akt	[51]
		Oral	Cancer Cells	Reduced DNA damage	[52]
miR-100-5p	miRNA	Lung	Cancer Cells	Targets mTOR	[57]
miR-193	miRNA	Esophageal	Cancer Cells	Cisplatin resistance	[55]
miR-196a	miRNA	Head and Neck	CAFs	CDKN1B and ING5 suppression	[54]
miR-423-5p	miRNA	Breast	Cancer Cells	Increased invasion and cisplatin resistance	[50]
miR-522	miRNA	Gastric	CAFs	Ferroptosis suppression	[58]

CAFs: cancer-associated fibroblasts, CDKN1B: cyclin-dependent kinase inhibitor 1B, circRNA: circular RNA, ING5: inhibitor of growth family member 5, miRNA: microRNA, mTOR: mammalian target of rapamycin, TAMs: tumor-associated macrophages.

**Table 3:** Exosome-mediated gemcitabine resistance.

Exosomal Cargo	Non-Coding RNA Type	Cancer	Exosomes Released From	Effect	Reference
miR-21	miRNA	Pancreatic	Cancer cells	Apoptosis suppression	[61]
miR-106b	miRNA	Pancreatic	CAFs	TP53INP1 inhibition	[62]
miR-146a	miRNA	Pancreatic	CAFs	Snail induction	[63]
miR-155	miRNA	Pancreatic	Cancer cells	Apoptosis suppression	[61]
miR-210	miRNA	Pancreatic	Stem cells	mTOR activation	[64]
miR-222-3p	miRNA	NSCLC	Cancer cells	SOCS3 regulation	[60]
miR-365	miRNA	Pancreatic	TAMs	Gemcitabine resistance	[59]

CAFs: cancer-associated fibroblasts, miRNA: microRNA, mTOR: mammalian target of rapamycin, NSCLC: non-small cell lung cancer, TAMs: tumor-associated macrophages.

**Table 4:** Exosome-mediated TKI resistance.

Exosomal Cargo	Non-Coding RNA Type	Cancer	TKI Affected	Effect	Reference
LNC000093	lncRNA	CML	Imatinib	TKI resistance	[79]
lncARSR	lncRNA	Renal	Sunitinib	miRNA sponging and AXL/c-MET targeting	[71]
miR-184	miRNA	NSCLC	Osimertinib	TKI resistance	[77]
miR-210	miRNA	NSCLC	Osimertinib	EMT induction	[76]
miR-549a	miRNA	Renal	Sorafenib	Increased angiogenesis through elevated HIF-1α and VEGF	[78]
miR-675	miRNA	CML	Imatinib	TKI resistance	[79]
miR-3913	miRNA	NSCLC	Osimertinib	TKI resistance	[77]

CML: chronic myeloid leukemia, EMT: epithelial–mesenchymal transition, lncRNA: long non-coding RNA, miRNA: microRNA, NSCLC: non-small cell lung cancer.

## Data Availability

Not applicable.
